# Bioluminescence Monitoring of Neuronal Activity in Freely Moving Zebrafish Larvae

**DOI:** 10.21769/BioProtoc.2550

**Published:** 2017-09-20

**Authors:** Steven Knafo, Andrew Prendergast, Olivier Thouvenin, Sophie Nunes Figueiredo, Claire Wyart

**Affiliations:** 1 Institut du Cerveau et de la Moelle épinière (ICM), Hôpital de la Pitié-Salpêtrière, Paris, France; 2 UMRS 1127, INSERM, Paris, France; 3 UMR 7225, CNRS, Paris, France; 4 Sorbonne Universités, Université Pierre et Marie Curie, Paris, France; 5 Assistance Publique des Hopitaux de Paris, Paris, France

**Keywords:** *GFP-aequorin-opt*, Zebrafish, Bioluminescence, Coelenterazine, Escape response

## Abstract

The proof of concept for bioluminescence monitoring of neural activity in zebrafish with the genetically encoded calcium indicator *GFP-aequorin* has been previously described (Naumann *et al*., 2010) but challenges remain. First, bioluminescence signals originating from a single muscle fiber can constitute a major pitfall. Second, bioluminescence signals emanating from neurons only are very small. To improve signals while verifying specificity, we provide an optimized 4 steps protocol achieving: 1) selective expression of a zebrafish codon-optimized *GFP-aequorin*, 2) efficient soaking of larvae in *GFP-aequorin* substrate coelenterazine, 3) bioluminescence monitoring of neural activity from motor neurons in free-tailed moving animals performing acoustic escapes and 4) verification of the absence of muscle expression using immunohistochemistry.

## Background

Unlike fluorescent genetically encoded calcium indicators (GECIs) (Grienberger and Konnerth, 2012), such as the GCaMP family, the bioluminescent indicator *GFP-aequorin* (Shimomura *et al*., 1962) does not require light excitation and therefore opens new avenues for monitoring neural activity in moving animals, including flies (Martin *et al*., 2007), mice (Rogers *et al*., 2007) and zebrafish larvae (Naumann *et al*., 2010). However, efficient use of *GFP-aequorin* remains challenging to achieve in zebrafish larvae, restricting its widespread use as a calcium indicator. The limitation lies in the fact that bioluminescence signals originating from a single muscle fiber are so large they constitute a major pitfall. Once absence of muscle expression is verified for a given transgenic line, bioluminescence signals emanating from neurons only are very small. To overcome these limitations, we developed a codon-optimized variant of *GFP-aequorin* for zebrafish larvae, achieved selective expression in motor and sensory neurons using existing transgenic lines, modified coelenterazine soaking protocol in order to conduct experiments at 4 days post-fertilization, created a behavioral bioluminescence assay for monitoring neuronal activity during acoustic evoked stereotyped escape responses in zebrafish larvae.

## Materials and Reagents

1. Microscope slide 76 x 26 x 1.1 mm (VINCENT LEERMIDDELEN SCIENTIFIC, catalog number: 29201316)
2. Cover glass Knittel Glass 24 x 60 mm
3. Zebrafish larvae Adult AB and and Tüpfel long fin (TL) strains of *Danio rerio* aged between 0 and 4 dpf (day post fertilization) were used for this study; transgenic lines used in this protocol (available on request): *Tg(mnx1:gal4)^icm11^;Tg(UAS:GFP-aequorin-opt)^icm09^*
4. Mammalian *GFP-aequorin* sequence (provided by Dr. Ludovic Tricoire, Université Pierre et Marie Curie, Paris, France, see text file in Supplement file 1)
5. PT2 14xUAS plasmid (provided by Pr. Koichi Kawakami, National Institute of Genetics, Mishima, Japan, map can be downloaded at http://kawakami.lab.nig.ac.jp/trans.html)
6. Instant Ocean^®^ salts
7. Methylene blue
8. Fixation solution (4% PFA) paraformaldehyde, powder 95% (Sigma-Aldrich, catalog number: 158127)
9. NGS (Abcam, catalog number: ab7481)
10. DMSO (Sigma-Aldrich, catalog number: D8418)
11. Triton X-100 (Sigma-Aldrich, catalog number: T9284)
12. Phosphate-buffered saline (PBS) (Thermo Fisher Scientific, Gibco™, catalog number: 18912014)
13. Chicken anti-GFP primary antibody (Abcam, catalog number: ab13970)
14. Alexa Fluor 488 goat anti-chicken IgG (Thermo Fisher Scientific, Invitrogen™, catalog number: A-11039)
15. Mounting Medium for fluorescence (Vector Laboratories, catalog number: H-1000)
16. Coelenterazine-h (Biotium, catalog number: 10111)
17. Propylene glycol
18. Cyclodextrine
19. Agarose
20. ‘Blue water’ (see Recipes)
21. Blocking solution (see Recipes)
22. Incubation solution (see Recipes)
23. Washing solution (see Recipes)

## Equipment

1. Wave generator (Agilent Technologies, catalog number: 33210A)
2. Audio amplifier (Lepai, catalog number: LP-2020A)
3. Upright confocal microscope (Olympus, model: FV1000)
4. 850 nm LED (Effisharp, Effilux, France, EFFI-sharp_CM_850_2)
5. Long-pass 780 filter (Asahi Spectra USA, catalog number: ZIL0780)
6. Long-pass 810 filter (Asahi Spectra USA, catalog number: XIL0810)
7. Diffuser (Thorlabs, catalog number: DG10-120-B)
8. High-speed infrared sensitive camera (Mikrotron, catalog number: Eosens MC1362)
9. Objective Nikkor 50 mm f/1.8D (Nikon, Japan)
10. Photomultiplier tube (PMT) (Hamamatsu Photonics, catalog number: H7360-02)
11. Acquisition card (National Instruments, catalog number: PCI 6602)
12. Band-pass filter (525 nm/50 nm) (ZEISS, catalog number: 489038-8002-000)
13. Short-pass filter (670 nm) (Asahi Spectra USA, catalog number: XVS0670)
14. 2-Ohm speaker
15. TTL chronogram generator (RD Vision, France, EG Chrono)
16. Black cardboard box protecting the setup from residual photons in the room
17. Standard equipment for molecular biology
18. Sonicator (Emerson Electric, Branson, model: B1510-DTH)

## Software

1. Hiris video software (RD Vision, France)
2. MATLAB (The MathWorks, catalog number: R2012b)

## Procedure

**Figure 1:**
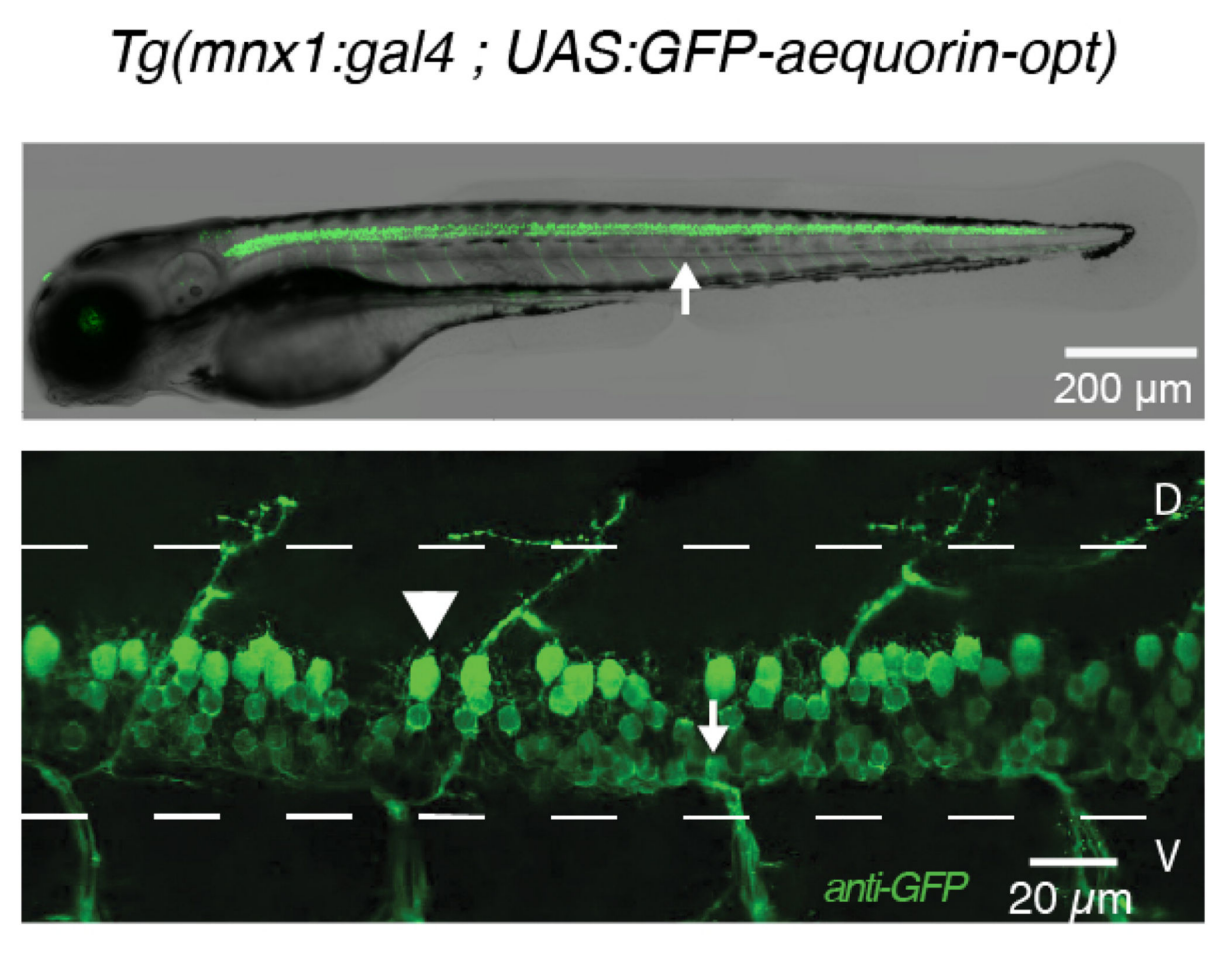
Expression pattern of *GFP-aequorin-opt* in motor neurons. Fluorescent image (upper panel) and immunohistochemistry for GFP (lower panel) in a 4 dpf *Tg(mnx1:gal4;UAS:GFP-aequorin-opt)* double transgenic zebrafish larva showing selective expression in spinal motor neurons (arrowhead: dorsal primary, arrow: ventral secondary motor neurons), and strictly no expression in muscle fibers (white arrow in the upper panel).

**Figure 2:**
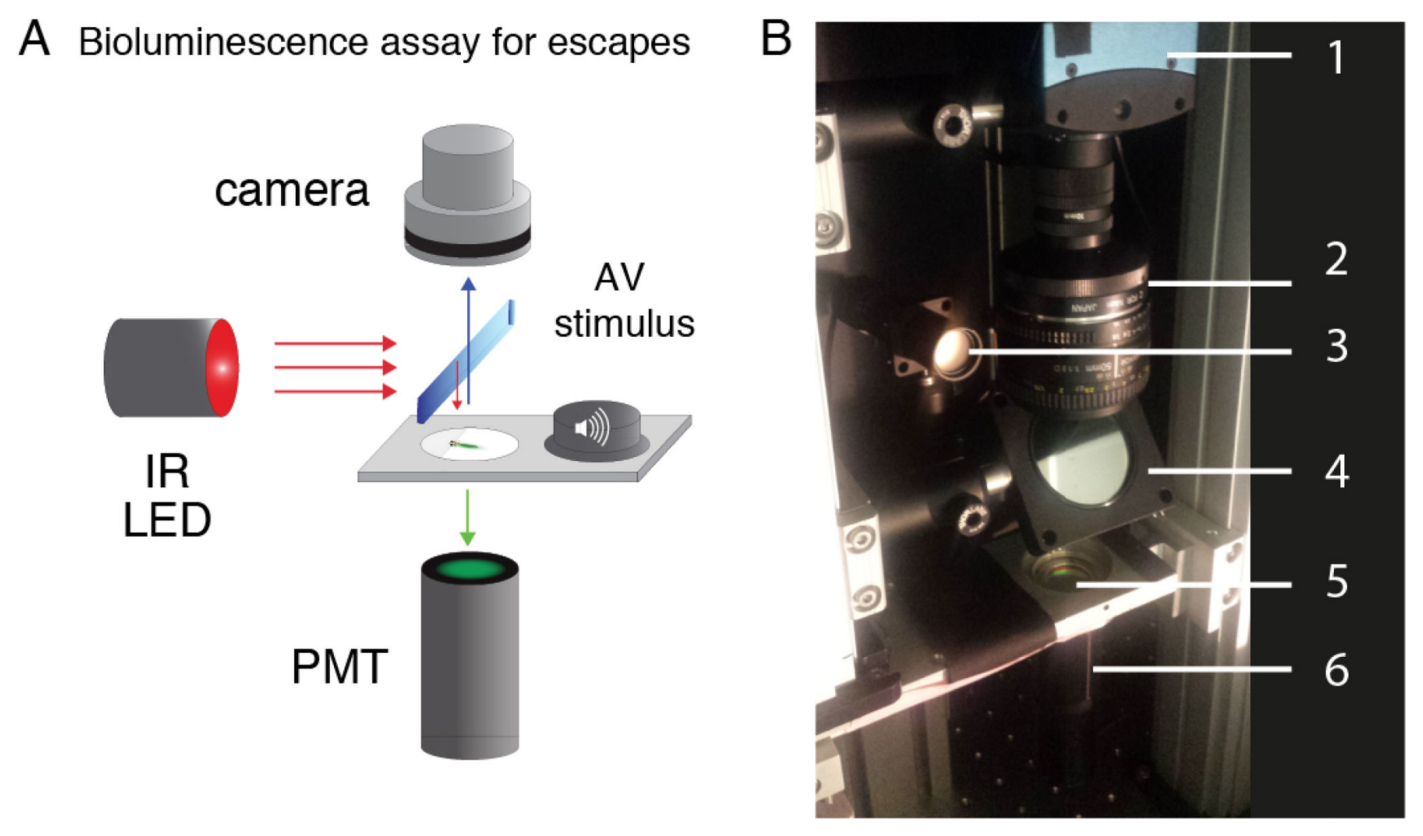
Bioluminescence setup for escapes. A. Signals emitted from spinal motor neurons in *Tg(mnx1:gal4;UAS:GFP-aequorin-opt)* double transgenic zebrafish larvae at 4 dpf were recorded using a photomultiplier tube under infrared illumination during active behaviors elicited by an acoustic stimulus. B. Picture of the setup showing every component: 1: high-speed camera; 2: 50 mm objective; 3: IR 850 nm LED; 4: Diffuser; 5: Long-pass filters; 6: photomultiplier tube.

- Generation of the codon-optimized *Tg(UAS:GFP-aequorin-opt*) transgenic line
  1. Generate a codon-optimized sequence, *GFP-aequorin-opt,* for expression in zebrafish from original sequence of mammalian *GFP-aequorin* (we used to online tool freely available made by Integrated DNA Technology: https://eu.idtdna.com/CodonOpt, original and optimized DNA sequences provided in Supplement file 2).
  2. Subclone *GFP-aequorin-opt* into a PT2 14xUAS plasmid.
  3. Inject the *UAS:GFP-aequorin-opt* construct in the *Tg(mnx1:gal4)^icm11^* embryos to generate the *Tg(mnx1:gal4;UAS:GFP-aequorin-opt)^icm09^* double transgenic line. Injection mix is composed as follows: 2 μl ADN (120 ng/μl) + 2 μl ARN transposase (175 ng/μl) + 1 μl KCl 2 M +1 μl 2% phenol red, complete to 10 μl with MilliQ H_2_O.
  4. Maintain adult AB and Tüpfel long fin (TL) strains of *Danio rerio* on a 14/10 h light cycle and water is maintained at 28.5 °C, conductivity at 500 μS and pH at 7.4.
  5. Raise embryos in ‘blue water’ (3 g of Instant Ocean^®^ salts and 2 ml of methylene blue at 1% in 10 L of osmosed water, see Recipes) at 28.5 °C during the first 24 h before screening for GFP expression.
- Characterization of *GFP-aequorin* expression with immunohistochemistry
  1. Fix 4 dpf larvae in 4% PFA for 4 h at 4 °C followed by 3 x 5 min washes in PBS.
  2. Block larvae for 1 h in blocking solution (see Recipes) (agitation required).
  3. Incubate larvae with the primary antibody (anti-GFP, dilution 1:500) over night at 4 °C in incubation solution (see Recipes) (agitation required).
  4. Wash three times for 5 min in washing solution (see Recipes), then incubate larvae in the dark with the secondary antibody (Alexa Fluor 488 goat anti-chicken IgG, dilution1:1,000) in PBST (agitation required) for 2 h at RT.
  5. Wash three times for 5 min in PBST, then mount larvae on a slide with mounting medium and image on a standard upright confocal microscope (Olympus FV-1000).
  6. Perform negative IHC controls by omitting the primary antibody.
  7. Image the entire immunostained *Tg(mnx1:gal4;UAS:GFP-aequorin-opt)^icm09^* larvae to confirm selective expression of *GFP-aequorin-opt* in spinal motor neuron populations and absence from muscle fibers. We noted more prominently primary dorsal motor neurons but also intermediate and ventral secondary motor neurons (Figure 1) without any expression in the muscles and only very limited expression in the brain and hindbrain.
- Soaking of larvae in coelenterazine solution
  1. Prepare 10 mM stock solution from lyophilized coelenterazine-h: *e.g*.,
    - For 250 μg of coelenterazine-h, final volume is 60 μl (M = 407.5 g/M).
    - Add propylene glycol to (25% of final volume, *e.g*., 15.4 μl).
    - Sonicate cyclodextrine at 45% (4.5 g in 10 ml).
    - Add cyclodextrine (75% of final volume, *e.g*., 46 μl).
  2. Prepare 60 μM coelenterazine-h soaking solution from stock:
    Dilute stock solution in ‘blue water’ (*e.g*., 6 μl in 1 ml for 10 embryos).
  3. Dechorionate embryos at 1 day post-fertilization under optical magnification.
  4. Soak dechorionated embryos overnight at 26 °C (*e.g*., 100 μl for each embryo, use 48-well plate sealed with paraffin).
  5. Renew 60 μM soaking solution at 2 days post-fertilization. Embryos are maintained in the dark.
  6. Perform behavioral experiments at 4 days post-fertilization (total soaking time is 72 h).
- Monitoring neuronal activity with bioluminescence
  1. Build a lightproof setup for bioluminescence assay (Figure 2)
    - Using black boards, create a 1 m square lightproof box.
    - Infrared light illumination is provided by an 850 nm LED mounted with 2 long-pass 780 and 810 filters and a diffuser.
    - Video acquisition is performed at 1,000 Hz using a high-speed infrared sensitive camera at 320 x 320 pixels resolution controlled by the video software (Hiris^®^).
    - Photons are counted with a photomultiplier tube located under the larva arena and sent to an acquisition card. A band-pass filter (525 nm/50 nm) and a short-pass filter (670 nm) are placed between the larva and the PMT.
    - A custom application-programming interface synchronizes the video acquisition with the photon count and the stimulus delivery using a 30 trials batched TTL chronogram.
  2. Run the bioluminescence assay one larva at a time
    - Place larva in a circular (2 cm diameter) 3D-printed arena (larva can also be head-embedded in 1.5% low-melting point agarose with the tail free to move).
    - Place the larva in the arena and attach the arena to a small 2-Ohm speaker.
    - Deliver sinusoidal stimuli (5 cycles, 500 Hz) produced by the waveform generator and audio amplifier through a 2-Ohm speaker attached to the larva arena.
    - Adjust intensity to the lowest value reliably eliciting an escape response (between 0.5 and 5 V usually).
    - Each trial consists in a 500 msec baseline followed by a 10 msec acoustic stimulus and 1,990 msec subsequent recording.
    - Assays consist of 30 trials with 1-min inter-trial intervals to reduce habituation.

## Data analysis

**Figure 3:**
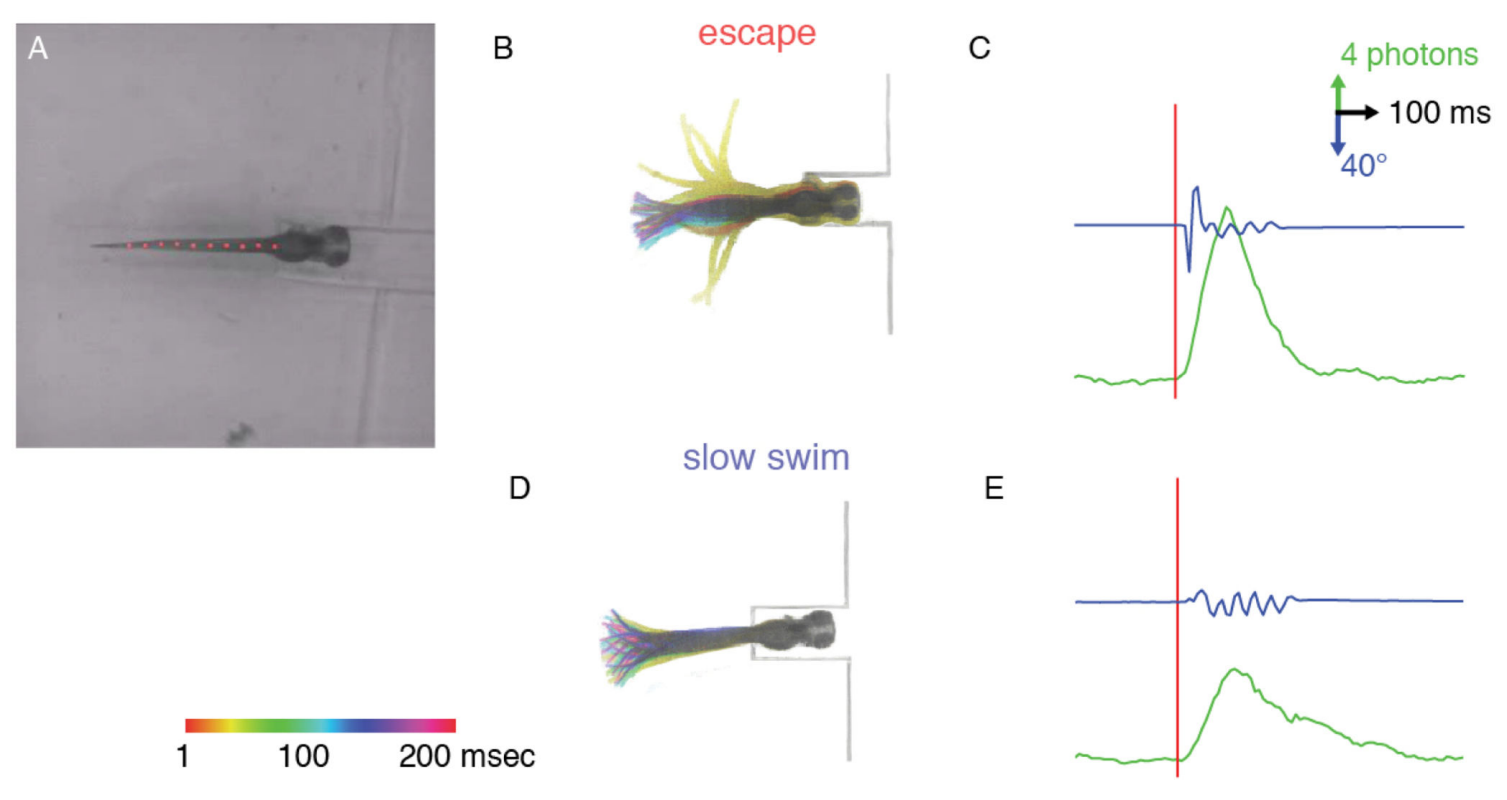
Example of kinematics and bioluminescence data for an escape response. A. 4 dpf larva in the head-embedded setup with the tail tracked (red dots) in transmitted IR light. B. Superimposed, and color-coded according to delay from stimulus, behaviors elicited by an acoustic stimulus; C. Example traces of typical bioluminescence signals and tail angle observed for an escape response; D. Example of a color-coded slow swim and (E) corresponding bioluminescence signal and tail angle traces for comparison with escapes.

- Kinematics analysis (blue trace in Figure 3, right panel)
  1. Using a custom MATLAB algorithm (see Supplement file 3), manually locate the base and tip of the tail. The tail is subsequently automatically tracked.
  2. The tail angle is computed for each frame and filtered using median filtering (window size = 10).
  3. The start of the movement is determined as the first frame followed by 3 with a differential tail angle value above 0.08 degree in our conditions.
  4. The end of the movement is determined as the last of 20 frames with a differential tail angle value below 0.1 degree in our conditions.
  5. Local minimal and maximal values of the tail angle occur at least 2 msec apart and 1° above the 5 msec preceding value.
  6. Automated movement categorization is determined as follows: ‘escapes’ for all movements with maximum values of tail angle > 45° and number of cycles > 1; ‘slow swims’ for all movement with maximum values of tail angle < 25° and number of cycles > 1.
- Bioluminescence recording and analysis (green trace in Figure 3B)
  1. Photons are counted at 1 kHz (temporal resolution of 1 msec) and then binned every 10 msec.
  2. The signal is filtered using a running average with a window size of 10, giving a typical signal-to-noise ratio (SNR) for active movements of 50 to 1.
  3. Noise is extrapolated from a linear fit of the cumulative photon count before the stimulus and subtracted from the signal.
  4. The start and end of the bioluminescent signal are computed respectively as the first time point followed by 3 points with a value above 0.4 photons/10 msec and below 0.2 photons/10 msec from this first point bioluminescence value.
  5. The time-to-peak is calculated between the start and the peak of the bioluminescent signal while the decay coefficient is derived from the one-term exponential fit between the peak and the end of the signal.

## Notes

1. Soaking in coelenterazine is key to obtaining a good signal-to-noise ratio for bioluminescence: pay attention to soaking conditions (incubation temperature, hour of coelenterazine renewal, keep the wells of the plate air-tight).
2. Noise due to ambient light must be tested before running the behavioral experiment; make sure the box is completely lightproof.
3. The *Tg(UAS:GFP-aequorin-opt)* zebrafish line is freely available to all users that request it.

## Recipes

1. ‘Blue water’
  3 g of Instant Ocean^®^ salts and 2 ml of 1% methylene blue in 10 L of ultrapure water
2. Blocking solution
  10% NGS
  1% DMSO
  0.5% Triton X-100
  in 0.1 M PBS
3. Incubation solution
  1% NGS
  1% DMSO
  0.5% Triton X-100
  in 0.1% PBS
4. Washing solution
  0.1 M PBST with 0.5% Triton X-100
